# Breast, cervical, and colorectal cancer screening rates amongst female Cambodian, Somali, and Vietnamese immigrants in the USA

**DOI:** 10.1186/1475-9276-8-30

**Published:** 2009-08-14

**Authors:** Ponnila S Samuel, Jane P Pringle, Nathaniel W James, Susan J Fielding, Kathleen M Fairfield

**Affiliations:** 1 University of Rochester School of Medicine & Dentistry (student), Rochester, NY, USA; 2 Department of Medicine, Maine Medical Center, Portland, Maine, USA; 3 Center for Outcomes Research and Evaluation, Maine Medical Center Research Institute, Portland, Maine, USA

## Abstract

**Introduction:**

Minority women, particularly immigrants, have lower cancer screening rates than Caucasian women, but little else is known about cancer screening among immigrant women. Our objective was to assess breast, cervical, and colorectal cancer screening rates among immigrant women from Cambodia, Somalia, and Vietnam and explore screening barriers.

**Methods:**

We measured screening rates by systematic chart review (N = 100) and qualitatively explored screening barriers via face-to-face questionnaire (N = 15) of women aged 50–75 from Cambodia, Somalia, and Vietnam attending a general medicine clinic (Portland, Maine, USA).

**Results:**

*Chart Review *– Somali women were at higher risk of being unscreened for breast, cervical, and colorectal cancer compared with Cambodian and Vietnamese women. A longer period of US residency was associated with being screened for colorectal cancer. We observed a 7% (OR 1.07, 95% CI 1.01–1.13, p = 0.01) increase in the odds that a woman would undergo a fecal occult blood test for each additional year in the US, and a 39% increase in the odds of a woman being screened by colonoscopy or flexible sigmoidoscopy for every five years of additional US residence (OR 1.39, 95% CI 1.21–1.61, p = 0.02). We did not observe statistically significant relationships between odds of being screened by mammography, clinical breast exam or papanicolaou test according to years in the US. *Questionnaire *– We identified several barriers to breast, cervical, and colorectal cancer screening, including discomfort with exams conducted by male physicians.

**Discussion:**

Somali women were less likely to be screened for breast, cervical, and colorectal cancer than Cambodian and Vietnamese women in this population, and uptake of colorectal cancer screening is associated with years of residency in this country. Future efforts to improve equity in cancer screening among immigrants may require both provider and community education.

## Introduction

In developing countries, incidence and mortality from cancer among women continues to be highest for breast and cervix cancers [1]. Stomach, lung, liver, esophageal, and colorectal cancers are also important causes of cancer-related mortality among women in less developed countries [1]. Among respondents to the National Health Institute Survey, women who immigrated to the US within the past ten years were less likely to be screened by mammogram, papanicolaou test (Pap), or fecal occult blood test (FOBT) than any other population group, including the uninsured [2]. Others have reported similar findings, including the importance of foreign birth place as a barrier [3], and wide ranging differences in knowledge and attitudes to screening among specific immigrant groups, particularly Cambodian and Vietnamese immigrants [4-7]. Cancer screening rates and barriers to screening among other Asian immigrants to Maine, and Somali women specifically, have not been well described. We sought to understand rates of screening tests for cervix, breast, and colorectal cancers among female immigrants receiving care in a primary care setting in Maine. Additionally, we sought to pilot a survey aimed at immigrant women to begin to understand barriers to adoption of recommended cancer screening tests for these women.

## Patients

We included female immigrants from Cambodia, Somalia, and Vietnam aged 50–75 in our study. Most new immigrant patients in our community have an initial intake health assessment in the International Clinic at Maine Medical Center (MMC). Cancer screening (*e.g*. clinical breast exam (CBE) and Pap test) occurs occasionally at this initial visit, depending on the patients' other health needs and their willingness to undergo a full examination. Otherwise, the patients are then referred to the Internal Medicine Clinic where primary care is provided by teams of attending physicians, resident physicians, nurse practitioners, and registered nurses. An electronic medical record has been in place since 2002.

## Methods

### Chart Review

We reviewed a total of 100 patient charts (85 patients chosen at random from the immigrant patient population in addition to 15 who were interviewed) during the calendar year 2005 in order to establish information about screening rates in the target populations. We selected the 85 charts at random from among a list of over 400 adult female patients from the countries of interest, aged 50–75. Inclusion criteria were immigration from one of the countries of interest, and having completed at least two visits to a physician or nurse practitioner in the clinic during the prior year. Patients were excluded if their country of origin was unclear, they had not had at least 2 visits to the clinic, or if they had been in the United States less than 1 year. Data systematically collected from chart reviews (via electronic medical records) included age, years of US residence, country of origin, marital status, personal or family history of breast, cervical, or colorectal cancer (CRC), and the year of the patient's most recent screening tests including Pap smear, CBE, mammography, colonoscopy or sigmoidoscopy, and FOBT.

### Questionnaire

We (one author – PS) administered a brief questionnaire (17 items, requiring 20–30 minutes to complete) to 15 patients via direct interview (some with and some without interpreter services, as deemed appropriate) in the patients' examination rooms after completion of a primary care appointment. Patients were invited to participate if they had a visit to their primary care physician during a 6 week period in summer of 2005 when we were able to have a research assistant (PS) available to administer the questionnaire. We did not collect data on number of women who were approached but declined, or reasons for declining. Physicians and nurse practitioners informed patients about this anonymous survey and invited the women to participate. We queried patients about their knowledge of breast, cervical, and colorectal cancers, respective screening options, patients' perception of preventive medicine, perceived barriers to screening, and patient preferences for receiving information on cancer screening. All women were also asked about the highest level of education they had received. Questionnaire results were assessed qualitatively by two authors (PS and KF). In some instances, it was not possible to do simple statistics (frequencies) to summarize questionnaire responses because patients were very resistant to choosing a specific category of response. Upon completion of the survey, the patients were provided packets on breast, cervical, and colorectal cancers written in their own languages (only breast cancer packets were provided for Somali women – no standardized, appropriate information about other malignancies was available at that time in Somali).

Interpreters (whomever had interpreted for the medical visit) included family relations, Catholic Charities of Maine (live agency interpreters), or contracted telephone interpreters.

We used SAS software packaging (SAS Institute, Cary, NC) to analyze collected chart review data using bivariable and multivariable logistic regression. In separate regression models, each screening test was the dependent variable, and age, country of origin, marital status, and years of US residency were potential independent variables. We report age-adjusted results for years of US residency since country of origin was collinear in that situation and marital status was not a confounder (it did not substantively change the effect estimates).

This study was approved by the Institutional Review Board at Maine Medical Center.

## Results

### Chart Review

**Figure 1:**
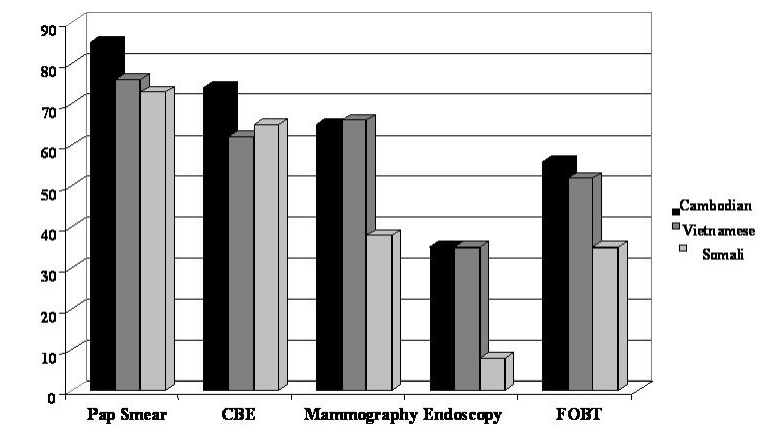
**Percentage of Immigrant Women Screened by Each Method, According to Country of Origin**. Pap indicates Papanicolaou test. CBE indicates clinical breast exam, Endoscopy refers to either colonoscopy or sigmoidoscopy, FOBT indicates fecal occult blood test.

Table 1 summarizes the patient population. The mean age of participating women was 60 years. Cambodian women were the earlier immigrants to the US, followed by the Vietnamese; Somali women were the most recent. For each screening procedure (Pap test, CBE, mammography, colonoscopy/sigmoidoscopy, and FOBT), we observed that Somali women were less often screened than Vietnamese and Cambodian populations (Figure 1).

**Table 1:** Patient Characteristics (Chart Reviews)

	Cambodian	Vietnamese	Somali
N (sample size)	34	29	37

Mean Age (range)	58 (50–75)	62 (50–75)	59 (50–73)

Years of US Residency (range)	14.7 (2–25)	9.2 (1–16)	5.5 (1–32)

In age-adjusted analyses with all women included, we observed a 7% (OR 1.07, 95% CI 1.01–1.13, p = 0.01) increase in the odds that a woman would undergo a FOBT for each additional year in the US. Similarly, we noted a 39% increase in the odds of a woman being screened by colonoscopy or flexible sigmoidoscopy for every five years of additional US residence (OR 1.39, 95% CI 1.21–1.61, p = 0.02). We did not observe statistically significant relationships between odds of being screened by mammography, CBE or Pap test according to years in the US. Due to sample size constraints, we were unable to examine these relationships by individual immigrant population.

Cambodian women had a greater chance of being screened for the studied malignancies than the other minorities, with the exception of breast cancer (for which Vietnamese women were more likely to be screened by mammography) (Figure 1). For example, 35% of the Cambodian groups studied were screened by colonoscopy/sigmoidoscopy versus 8% of Somali women. Notably, these are simple frequencies and are therefore not adjusted for duration of time in the US.

### Questionnaire

Of the 15 respondents, 9 were Cambodian, 4 were Vietnamese, and 2 were Somali. The average duration of school attendance was 3 years. Most women reported that they knew "nothing" or "little" about breast, cervical, or colorectal cancer, but many had difficulty answering the question because they were not sure about choosing a specific category of response. Regardless of this fact, however, 13/15 participants supported the idea of cancer screening for people over 50, after being provided with a definition of cancer screening.

The question, "What might prevent you from being screened for cancer?" was asked, with the following possible responses (participants could respond yes to as many as applied): a). inability to pay; b). lack of transportation to doctor's office; c). fear or embarrassment; d). dislike of having a male physician perform the examination; e). cannot speak English/cannot speak English well; f). cannot read/cannot read English well; g). process is too uncomfortable/risky. Reported barriers preventing these women from being screened for any cancer included all categories described above, with "discomfort with a male provider" being one of the most common barriers reported.

When participants were asked how they would like to learn more about cancer screening, the two most popular choices were 1). "Written information to read in your own language" and 2). "Videos in your own language that explain the screening process".

## Discussion

We observed fair rates of breast and cervical cancer screening, and low screening rates for CRC among immigrant women. Cambodian women were more likely to be screened for each malignancy than Vietnamese women, who, in turn, were more likely to be screened than the Somalis. Longer duration of time in the US was related to improved screening rates. In our population, most Cambodians have resided in the US for an average of 15 years, while the Vietnamese and Somalis have been residing in the country for an average of 9 and 6 years, respectively.

Our rates for the Asian immigrants are similar to West Coast studies on Vietnamese and Cambodian immigrants [8] and to those reported among Hispanics in the US and Puerto Rico [9]. Prior research on cancer screening among immigrants suggests that language barriers, low educational attainment, and poverty lead to decreased access to preventive care [10-12]. In our studied population, increased discomfort of immigrant women with male providers was a reported barrier, however, this barrier was ascertained by our pilot questionnaire (with limited possible responses) rather than an empiric qualitative method such as focus groups, where patients may have offered other barriers to screening. Similar observations of non-English speaking immigrant women declining examination by male physicians, and other cultural factors acting as barriers, may contribute to low screening rates [12].

We found that among the screening tests studied, colonoscopies/sigmoidoscopies were the least performed examination for immigrant women, similar to findings in other studies [3,13]. In our review of charts, we observed health care providers were often unable to discuss screening due to other dominating issues. This problem of "tyranny of the urgent" has been described in well known work attempting to improve the consistency of preventive care and care of chronic illnesses [14,15]. The process for requesting an endoscopy procedure is also more complex than that for a Pap test or a CBE, which can be done by the patient's own provider. Additional appointments must be set up with translators to explain the endoscopy preparatory procedures, which, in turn, must be done correctly in order to obtain valid results. If the patient misses these appointments or the actual procedure itself, the probability of completing the screening test decreases. Moreover, the concept of disease states (*e.g*. cancer) and of preventative care, in general, may be difficult for immigrants, and cultural health beliefs may influence choices even with health care education. These observations highlight the importance of enhanced systems of care for immigrant populations. Improvement of patient health care via increased physician-patient communication, professional interpreters, cultural understanding, counseling about gender bias, and awareness of patient health beliefs are needed for high quality preventive health care.

This study was limited by its small sample size. Our data suggested trends that could be confirmed only with a larger sample, and ideally at more than one center. Local factors may also have influenced our findings. For example, a recent educational project in the Vietnamese community in the Portland area may have raised breast cancer screening rates in that population locally. We did not assess the role of insurance status, as the vast majority of this population was covered by Medicaid. An additional limitation is that we did not explore problems with access to health care such as rides, work, or childcare constraints. Patient surveys were also conducted with mostly Cambodian and Vietnamese women. For unclear reasons (perhaps chance), there were few Somali women having clinic visits during enrollment. Of those that were seen in the Internal Medicine Clinic, many did not meet entry requirements, while others declined participation; as a result, only two Somali women were interviewed. Additionally, many respondents had difficulty choosing a single response to the questions. Future work might include focus groups or other methodology rather than questionnaires to gather information on themes related to understanding of cancer and cancer screening. This would also avoid bias that may have been introduced by the questionnaire responses (such as that having a male provider was a problem).

We report inadequate cancer screening rates among women immigrants, specifically Somalis, receiving care at our medical center. Screening rates were highest among Cambodian women, who had been in the US the longest. If duration of residence in the US is a proxy for acculturation, this is likely to explain our findings. Most of the (albeit small) sample of immigrant women we spoke to were genuinely interested in learning about cancer screening with written information and videos in their own languages (low levels of education among these women may decrease use of written information). Additional detailed assessment of immigrant understanding of cancer screening may be better approached using a qualitative method such as focus groups. Examination of the various belief systems by cultural group in this setting would allow for exploration of a wider variety of potential beliefs, understanding levels, and barriers to full participation in medical care in the US. Additional studies could assess the value of "screening days" (which appeared to have been successful in the case of Vietnamese women and mammography screening in our community) which could be held for specific groups of immigrants with involvement of community leaders and in-person professional interpreters. These would provide women with access to female health care providers, cultural liaisons, and interpreters in a setting that promotes cultural awareness. In the case of Somali immigrants, additional work is needed to understand health beliefs and to engage the population in cancer screening. The first steps again may need to be focus groups or in-depth interviews. Working with religious leaders or community leaders around educational interventions for the community may also be helpful. Additional research into provider bias or perceptions of cancer risk and screening adherence and ability in immigrants would also extend this field of inquiry. To improve colon cancer screening among immigrant populations, more intensive educational efforts may be necessary, regardless of ethnicity. In conclusion, we suggest several areas for study and community work that may help improve cancer screening rates among immigrants.

## Competing interests

The authors declare that they have no competing interests.

## Authors' contributions

PS participated in the study design, carried out data collection and entry, and was responsible for the first draft of the manuscript. JP participated in the study design and coordination, partipated in data review and interpretation, and assisted witih manuscript revisions. NJ participated in the study conception and design, assisted with data interpretation, and assisted with manuscript revisions. SF participated in the study design, assisted with interpretation of the qualitative interviews, and assisted with manuscript revision. KF lead the study team, designed the study, carried out statistical analyses, and participated in the manuscript first draft and revisions. All authors read and approved the final manuscript.
